# A Novel Tetrapeptide Derivative Exhibits In Vitro Inhibition of Neutrophil-Derived Reactive Oxygen Species and Lysosomal Enzymes Release

**DOI:** 10.1155/2013/853210

**Published:** 2013-05-30

**Authors:** Sumitra Miriyala, Manikandan Panchatcharam, Meera Ramanujam, Rengarajulu Puvanakrishnan

**Affiliations:** ^1^Department of Biotechnology, Central Leather Research Institute, Chennai 600020, India; ^2^Department of Cellular Biology and Anatomy, Louisiana Health Sciences Center, Shreveport, LA 71130, USA; ^3^Immunology and Inflammation, Boehringer Ingelheim Pharmaceuticals, Inc., Ridgefield, CT 06877, USA

## Abstract

Neutrophil infiltration plays a major role in the pathogenesis of myocardial injury. Oxidative injury is suggested to be a central mechanism of the cellular damage after acute myocardial infarction. This study is pertained to the prognostic role of a tetrapeptide derivative PEP1261 (BOC-Lys(BOC)-Arg-Asp-Ser(tBu)-OtBU), a peptide sequence (39–42) of lactoferrin, studied in the modulation of neutrophil functions in vitro by measuring the reactive oxygen species (ROS) generation, lysosomal enzymes release, and enhanced expression of C proteins. The groundwork experimentation was concerned with the isolation of neutrophils from the normal and acute myocardial infarct rats to find out the efficacy of PEP1261 in the presence of a powerful neutrophil stimulant, phorbol 12-myristate 13 acetate (PMA). Stimulation of neutrophils with PMA resulted in an oxidative burst of superoxide anion and enhanced release of lysosomal enzymes and expression of complement proteins. The present study further demonstrated that the free radicals increase the complement factors in the neutrophils confirming the role of ROS. PEP1261 treatment significantly reduced the levels of superoxide anion and inhibited the release of lysosomal enzymes in the stimulated control and infarct rat neutrophils. This study demonstrated that PEP1261 significantly inhibited the effect on the ROS generation as well as the mRNA synthesis and expression of the complement factors in neutrophils isolated from infarct heart.

## 1. Introduction

Reactive oxygen species (ROSs) have been shown to exert a direct inhibitory effect on myocardial function in vivo and have a critical role in the pathogenesis of myocardial stunning [1, 2]. Oxidative stress and formation of ROS could set off a cascade of biochemical and molecular squeal such as the xanthine dehydrogenase/xanthine oxidase conversion, leading to over production of ROS [3, 4]. Oxidative ischemic injury is suggested to be a central mechanism of the cellular damage affecting all organs and tissues after ischemia; however, the mechanisms, which trigger and modulate this damage, have not been fully characterized. 

Polymorphonuclear leukocytes (PMNLs) are short-lived, terminally differentiated cells that act against all infections and they are one of the most important cellular components involved in host defense. Circulating PMNLs participate in host defense by margination and extravasations at the site of inflammation [5]. Although neutrophils are essential to host defense, they have also been implicated in the pathology of ischemia [6, 7] and in many chronic inflammatory conditions [8, 9]. Neutrophil levels are activated in myocardial infarction [10], and subsequently, activated neutrophils produce reactive oxygen species (ROS) such as superoxide anion (O_2_^∙−^), hydrogen peroxide (H_2_O_2_), hypochlorous acid (HOCl), and possibly hydroxy radical (OH^∙^) [11, 12]. 

 Therefore, the accumulation of oxygen free radicals and activation of neutrophils are strongly implicated as important pathophysiological mechanisms mediating myocardial ischemia [2, 13]. Thus, the site of inflammation is characterized by a high concentration of stimulated neutrophils, which secrete ROS and proteolytic enzymes [14–16].

Complement activation constitutes facet of inflammation, which occurs during ischemia [17–19]. A variety of entities activate complement, including antibodies, membranes of microorganisms, and free radicals [20]. Although it is known that free radicals activate the complement system, the effect of free radicals on complement transcription remains unexplained.

In the present work, we identified that PEP1261 [21] could inhibit the ROS and lysosomal enzymes release from activated neutrophils isolated from acute myocardial infarct rats [1]. 

## 2. Materials and Methods

This study conforms to the guiding principles of Institutional Animal Ethics Committee (IAEC), Committee for the Purpose of Control and Supervision of Experiments on Animals (CPCSEA), and the Guide for the Care and Use of Laboratory Animals published by the National Institutes of Health (NIH Publication no. 85–23, revised 1996).

### 2.1. Chemicals

Phorbol 12-myristate 13 acetate (PMA), cytochrome C, superoxide dismutase, phenol red, dextran, O-dianisidine hydrochloride, ficoll-histopaque (Sigma 1077), glycerol, hexadecyltrimethylammonium bromide, Hank's balanced salt solution (HBSS), horse radish peroxidase, and Triton X100 were purchased from Sigma (St. Louis, MO, USA). Folin-Ciocalteau reagent, hemoglobin, hydrogen peroxide, and p-nitrophenol phosphate were obtained from Sisco Research Laboratories (Bombay, India). All other chemicals used were of analytical grade.

### 2.2. Experimental Animals

Female rats (Wistar) weighing 180–200 g were inbred in a pathogen free facility, and they were maintained in environmentally controlled rooms with 12 h light/dark cycle. The animals received commercial rat diet and water ad libitum.

### 2.3. Synthesis of PEP1261

**Figure 1:**
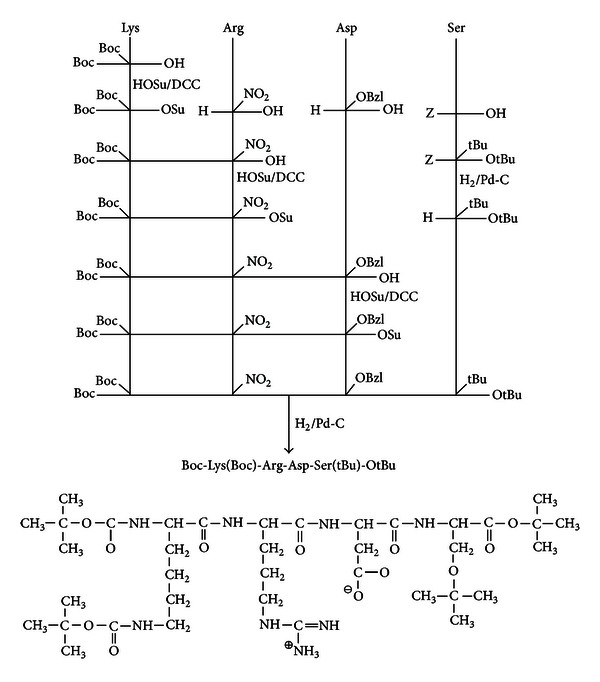
Scheme of synthesis of PEP1261 with its structure.

 PEP 1261 used in this study was synthesized by solution phase methodology as represented schematically in Figure 1 and purified by column chromatography. The homogeneity of the peptide was established by thin layer chromatography. Proton NMR spectra and IR spectra were recorded using Brucker 300 MHz FT-NMR spectrometer and Nicolet DX-20 FT-IR spectrometer, respectively. Amino acid analysis of the peptide derivative was performed by precolumn derivatization with phenylisothiocyanate (PITC) using HPLC. Reverse phase HPLC separation of PTC amino acids was performed with Pharmacia LKB LCC 2252/LKB VWM 2141 unit with fixed or variable wavelength detector at 254 nm. DuPont Zorbax PTH column, 25 cm in length, 0.46 cm internal diameter, and 5 *μ*m particle size, was used. The FAB mass spectra were recorded on a JEOL SX 102/DA-6000 Mass Spectrometer Data system [21].

### 2.4. Development of the Infarct Model

Myocardial infarct model was induced in rats as described earlier [1] with modifications to minimize the early mortality rate. Briefly, the animals were given a mucus secretor blocker glycopyrrolate [2 *μ*g/kg.b.wt., i.m) and were anaesthetized with ketamine [50 mg/kg.b.wt., i.p] and diazepam (2.5 mg/kg.b.wt., i.p]. After anesthesia, endotracheal intubation was performed and a tube connected to a positive pressure respirator was introduced. After establishing positive pressure respiration, left intercostal thoracotomy was performed using aseptic technique, and the third and fourth intercostal ribs were separated with a small retractor to expose the heart. The pericardium was opened carefully avoiding any injury to the heart. The left coronary artery (LCA) and its branches could be seen easily without any amplification or use of a surgical microscope. The pattern of the LCA was carefully examined in order to ligate the left anterior descending coronary artery. A 6–0 atraumatic proline silk suture was passed through the epicardial layer around the midway of the left anterior descending coronary artery. Following coronary occlusion, the thorax was closed in layers, the endotracheal tube was removed, and the animals were brought back to normal respiration.

### 2.5. Isolation of Neutrophils from Rats

Blood was drawn from sham-operated and infarct rats, and neutrophils were separated according to Newman et al. [22]. The neutrophils were suspended in HBSS, and the cell concentration was determined using a haemocytometer. 

### 2.6. Stimulation Studies

To ascertain the optimal concentration of PEP1261 on neutrophils isolated from rats, myeloperoxidase (MPO) was used as a sensitive marker and the results showed an inhibitory concentration (IC_50_) of 94.10 ± 8.38 *μ*M (data not shown). Hence, further studies were carried out with an optimal concentration of 120 *μ*M for PEP1261 and KRDS. Neutrophils (1 × 10^6^ cells/well) were left for adherence for 1 h, and they were stimulated by the addition of PMA (100 ng/mL) [23] for 1 h at 37°C. In the case of sham-operated and infarct rat, neutrophils were isolated and after 1 h adherence and PEP1261 was added. The culture was terminated after 1 h of PEP1261 treatment. 

MPO was assayed in the cell lysate after extracting the enzyme in phosphate buffer containing hexadecyl trimethylammonium bromide [24].

For H_2_O_2_ assay [25], the medium was removed and to the adherent neutrophils, 100 *μ*L (1 *μ*g/mL) of PMA was added followed by the addition of 1000 *μ*L of HBSS containing horse radish peroxidase (19 units/mL) and phenol red (0.02%). After 1 h of incubation, 100 *μ*L of 1 M NaOH was added and the color developed was read at 605 nm.

For the assay of O_2_^∙−^ [26], the medium was removed and the adherent neutrophils were incubated for 1 h in HBSS containing 80 *μ*M cytochrome C and 10 *μ*M PMA. The color developed in the supernatant was read at 580 nm. The amount of O_2_^∙−^ released was measured by the amount of cytochrome C using a molar extinction coefficient of 21 × 10^3^ M^−1^ cm^−1^.

For the lysosomal enzyme assays, after removing the cell free medium, the cells were lysed by adding 0.5 mL of ice cold 0.1% Triton X100 in 0.25 M sucrose, subjected to repeated freezing and thawing and assayed for acid phosphatase [27] and cathepsin D [28].

### 2.7. Complement Expression Study

Total RNA from neutrophils isolated from sham operated and infarct tissue was extracted using Trizol reagent (kit supplied by Gibco-BRL (Life technologies) (US Patent no. 5,346,94)) a monophasic solution of phenol and guanidine isothiocyanate developed by Chomczynski and Sacchi [29] (Table 1).

### 2.8. Statistical Studies

Univariate analysis was carried out for all the parameters, and the results were analyzed by nonparametric statistics Mann-Whitney “*U*” test.

## 3. Results

**Figure 2:**
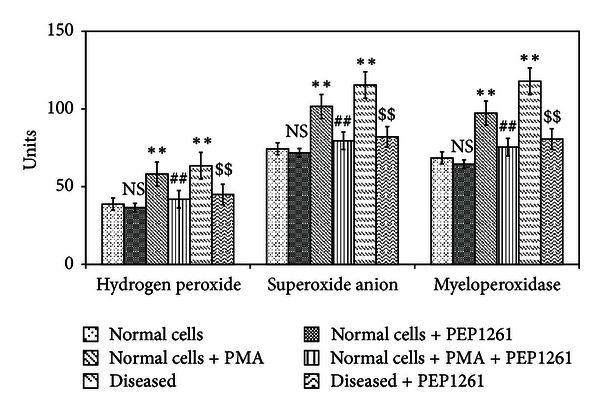
Effect of PEP1261 on myocardial infarct rat neutrophils ROS generation. All values are mean ± SD (*n* = 20). H_2_O_2_ level was expressed as *μ*moles of H_2_O_2_ liberated/0.5 × 10^6^ cells. O_2_^∙−^ level was expressed as nmoles of O_2_^−^ liberated/min/mg protein. MPO level was expressed as *μ*moles of H_2_O_2_ utilized/min/mg protein. NS = nonsignificant as compared to control; **P < 0.01 as compared to control; ^##^ *P* < 0.01 as compared to PMA stimulated cells; ^$$^ *P* < 0.01 as compared to rat myocardial infarct cells.

Studies on PMA stimulated rat neutrophils showed the beneficial effect of PEP1261 on the ROS generation and lysosomal enzymes release, and, hence, experiments were performed on neutrophils isolated from myocardial ischemic rats. While the levels of H_2_O_2_ and O_2_^∙−^ were significantly increased in both PMA stimulated and in the neutrophils isolated from myocardial infarct rats, PEP1261 showed a protective effect towards free radical generation, irrespective of the nature of the stimulant (*P* < 0.01) (Figure 2). 

In comparison with PMA treated cells, PEP1261 cells demonstrated a significant decrease in PMA-induced MPO activation (*P* < 0.01). Consistent with these observation, neutrophils derived from myocardial infarct rat were significantly inhibited upon PEP1261 treatment (*P* < 0.01). 

**Figure 3:**
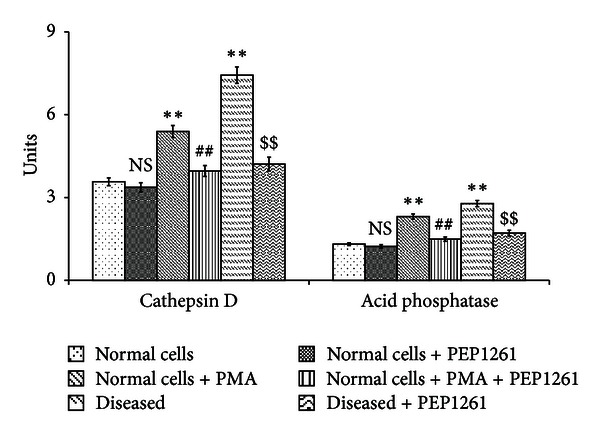
Effect of PEP1261 on myocardial infarct rat neutrophils lysosomal release. All values are mean ± SD (*n* = 20). Acid phosphatase activity was expressed as *μ*moles of p-nitrophenol liberated/h/mg protein. Cathepsin D activity was expressed as *μ*moles of tyrosine liberated/h/mg protein. NS = nonsignificant as compared to control; *P < 0.05 as compared to control; ^#^ *P* < 0.05 as compared to PMA stimulated cells; ^$^ *P* < 0.05 as compared to rat myocardial infarct cells.

Lysosomal enzymes release in response to PMA was also enhanced in neutrophils and was corrected by treatment with PEP1261 as shown in Figure 3. PMA stimulation resulted in a moderate increase (*P* < 0.05) in the levels of cathepsin D and acid phosphatase, and the levels of these enzymes were noticed to decrease upon treatment with PEP1261 (*P* < 0.05).

**Figure 4:**
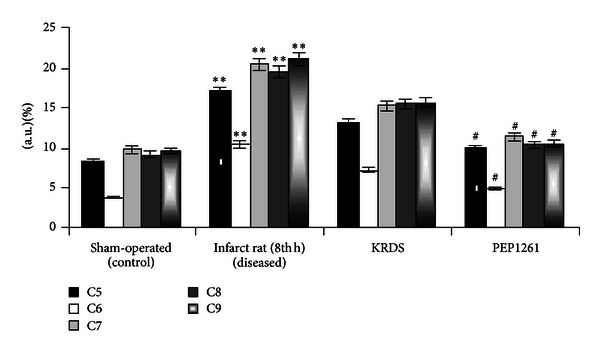
Densitometry analysis of the functional complement factor proteins. All values are mean ± SD (*n* = 6). **P < 0.01 as compared to control and ^#^ *P* < 0.05 as compared to diseased (8th hour infarct myocardium) using post hoc tukey's test.

 To ensure that the results obtained are from the neutrophils derived from the acute myocardial infarcted rats, RT-PCR amplification from total RNA extracts was used to establish the presence and relative values of the mRNAs for the functional terminal C proteins, namely, C5, C6, C7, C8, C9, and GAPDH (Figure 4). 

## 4. Discussion

Neutrophils represent the first line of host defense against all types of infection, and they are also involved in the pathology of various inflammatory conditions [30]. The ability to survive an infection challenge might depend on the appropriate modulation of these neutrophil functions [31]. 

 Oxidative stress, defined as an increase in the production of ROS, namely, O_2_^∙−^, H_2_O_2_, and OH^∙^ has been related to reperfusion injury in heart and other organs [32, 33]. Neutrophils recruitment depends on the presence of inflammatory mediator. These cells, therefore, may exacerbate tissue injury through the release of free radicals and proteolytic enzymes. The oxygen metabolites are produced by a membrane bound enzyme complex, the NADPH oxidase. Thus, in ischemic conditions, there is always the adhesion and activation of neutrophils with the generation of free radicals. Hence, the development of tissue injury depends upon the balance between the generation of ROS and tissue antioxidant status [34]. Any disturbance in this equilibrium in favor of free radicals causes an increase in oxidative stress and initiates subcellular changes leading to cardiomyopathy and heart failure [10].

Several drugs were found to curtail these deleterious effects, and of recent interest is the role of sequence specific peptides as therapeutic agents in many disorders. The sequence H-Lys-Arg-Asp-Ser-OH (KRDS) is an analog of RGDS and corresponds to residues 39–42 of human lactoferrin. It is well known that human lactoferrin exhibits anti-inflammatory and antimicrobial properties, and it has a role in regulating various components of the immune system, growth factor activity, and in inhibiting platelet aggregation [35]. This sequence, situated near the N-terminal region of the protein and known to inhibit platelet aggregation in vitro conditions, was suitably modified with hydrophobic groups (PEP1261) to enhance the permeability across the membrane and also to increase the stability. 

Preliminary studies were carried out using PMA stimulated human neutrophils, and PEP1261 was found to significantly inhibit ROS species generation and lysosomal enzymes release at a concentration of 120 *μ*M. The concentration of 120 *μ*M did not seem to be very high since earlier experiments showed that the peptide inhibited platelet aggregation only after critical micellar concentration (CMC) (60 *μ*M) was achieved and a concentration above 153 *μ*M was found to effectively inhibit platelet aggregation [21]. 

MPO activity, a marker of leukocyte accumulation, was markedly elevated in heart injury. It was shown recently that the neutrophils characteristically invaded the myocardial tissue during ischemia and they were observed to be the major source of free radicals [2]. The result of this study showing PEP1261 significantly reducing O_2_^∙−^, H_2_O_2_, and MPO levels both in the case of stimulated and myocardial rat infarct neutrophils was also in agreement with that of Manikandan et al. [36]. 

The extensive release of O_2_^∙−^ derivatives was probably involved in the pathogenesis of tissue damage following ischemia in stroke and myocardial infarction and in respiratory distress syndrome [35]. Ischemia induced an acute inflammatory response in myocardial tissue with an early phase of neutrophil accumulation, which was accelerated by reperfusion. The rise in H_2_O_2_ level during myocardial ischemia indicated that intensification of oxygen free radical production occurred. This might be the result of hypoxanthine conversion by xanthine oxidase, catecholamine auto-oxidation, polymorphonuclear neutrophil activation, and/or derangement within mitochondrial electron transfer [37]. In experimental models, interventions that depleted neutrophils or inhibited their function caused a significant reduction in myocardial infarct size [36].

Lysosomal enzymes could completely degrade the components of connective tissue such as collagen, protein-mucopolysaccharide complexes, glycoproteins, and elastin [38]. These observations concurred with our findings that there was an increased activity of lysosomal enzymes such as acid phosphatase and cathepsin D in myocardial ischemic neutrophils which were well brought under the control levels upon PEP1261 treatment.

In summary, neutrophil-mediated ROS, as well as lysosomal enzyme leak in particular, increases after injury. Treatment with PEP1261 attenuates ROS generation and may attenuate cardiac inflammation. We propose that these observations reflect a role for complement mediated activation of neutrophil in acute myocardial infarction. Our observation may suggest novel strategies to attenuate inflammation during cardiac injury and suggest that a better understanding of inflammatory cells and environmental stimuli that alter ROS levels is warranted.

## Figures and Tables

**Table 1:** 

Gene	Sequence of primer (Sense)	Sequence of primer (Antisense)	Product length (base pair)
C5	CAGCATAATTCAGGGTGAACG	CAGCTTGTCATTTGAGCCAC	315
C6	TGCAGTGACAAAACGGAACAACCTC	TGCAGTCTTCCTCTTGTCGCTTCTC	338
C7	GGAACAGAGTCAATACCAAAAG	ACTGCGTGAAGAAGATGATGAAGAT	248
C8	GACTGCGACCCTCTTGACTCTGCTC	TTTCGGAAGGTACTGACAGCCATGG	258
C9	GAATGAGCCCCTGGAGTGAATGGTC	CATTTCCGCAGTCATCCTCAGCATC	316
